# Force produced after stretch in sarcomeres and half-sarcomeres isolated from skeletal muscles

**DOI:** 10.1038/srep02320

**Published:** 2013-07-31

**Authors:** Fábio C. Minozzo, Bruno M. Baroni, José A. Correa, Marco A. Vaz, Dilson E. Rassier

**Affiliations:** 1 Department of Kinesiology and Physical Education, McGill University, Canada; 2 School of Physical Education, Federal University of Rio Grande do Sul, Brazil; 3 Department of Mathematics and Statistics, McGill University, Canada; 4 School of Physical Education, Federal University of Rio Grande do Sul, Brazil; 5 Departments of Kinesiology and Physical Education, Physiology and Physics, McGill University, Canada

## Abstract

The goal of this study was to evaluate if isolated sarcomeres and half-sarcomeres produce a long-lasting increase in force after a stretch is imposed during activation. Single and half-sarcomeres were isolated from myofibrils using micro-needles, which were also used for force measurements. After full force development, both preparations were stretched by different magnitudes. The sarcomere length (SL) or half-sarcomere length variations (HSL) were extracted by measuring the initial and final distances from the Z-line to the adjacent Z-line or to a region externally adjacent to the M-line of the sarcomere, respectively. Half-sarcomeres generated approximately the same amount of isometric force (29.0 ± SD 15.5 nN·μm^−2^) as single sarcomeres (32.1 ± SD 15.3 nN·μm^−2^) when activated. In both cases, the steady-state forces after stretch were higher than the forces during isometric contractions at similar conditions. The results suggest that stretch-induced force enhancement is partly caused by proteins within the half-sarcomere.

When an activated muscle is stretched during activation, the force that is being produced increases significantly. After the stretch, the force declines but remains higher than that produced during isometric contractions at corresponding lengths^1^. The mechanism behind this long-lasting increase in force is unknown. Sarcomere length non-uniformities^2^,^3^ that develop during activation and after stretch^4^ have been commonly used to explain the residual force enhancement, but this hypothesis has been challenged by studies performed with isolated myofibrils, which allow tracking of individual sarcomeres^5^,^6^, and more tellingly by studies performed with individual sarcomeres^7^,^8^. The latter are particularly important; it was observed that single sarcomeres produce force enhancement when stretched during activation^8^.

In one of these studies^8^, significant A-band displacements toward one of the sides of the sarcomeres during activation and stretch were observed, corroborating previous findings showing non-uniform behavior of half-sarcomeres in myofibrils during contractions^9^ and after stretch^10^. The amount of A-band displacement was strongly correlated to the levels of force enhancement^8^, suggesting that half-sarcomere length could play a role in force enhancement. Accordingly, one of the half-sides of the sarcomere would be stronger after stretch due to an increase in filament overlap, while titin would be strained in the other half, ultimately contributing to the overall extra gain in force. In fact, a recent model that simulated force behaviour after stretch considering non-uniform half-sarcomeres was able to predict force enhancement at levels that were similar to those observed experimentally – between 2% and ~13%^11^.

The next logical step in the evaluation of the mechanisms behind the stretch-induced force enhancement is evaluating the effects of stretch on half-sarcomeres, which has been impossible so far due to technical limitations. We developed a technique that allows, for the first time, experiments to be performed with mechanically isolated half-sarcomeres. Our first goal was to test if half-sarcomeres would reliably contract when activated, generating levels of force in line with those previously reported in larger preparations. Our second goal was to test if half-sarcomeres would produce an increase in force during stretch, and if the force would remain elevated after the end of the stretch. The latter would indicate that the residual force enhancement commonly seen in larger preparations is a phenomenon associated with the half sarcomere, a preparation where A-band movements during activation and stretch are prevented.

## Results

Isolated half-sarcomeres generated approximately the same amount of force on average (29.0 ± 15.5 nN·μm^−2^, n = 17) as single sarcomeres (32.1 ± 15.3 nN·μm^−2^, n = 18) during isometric contractions (p = 0.6). These levels of force are slightly lower than previously reported in single sarcomeres^7^,^8^; the difference may be due to temperature, as the current study used 10°C, lower than previous studies that used 15°C^7^ or 20°C^7^,^12^.

**Figure 1:**
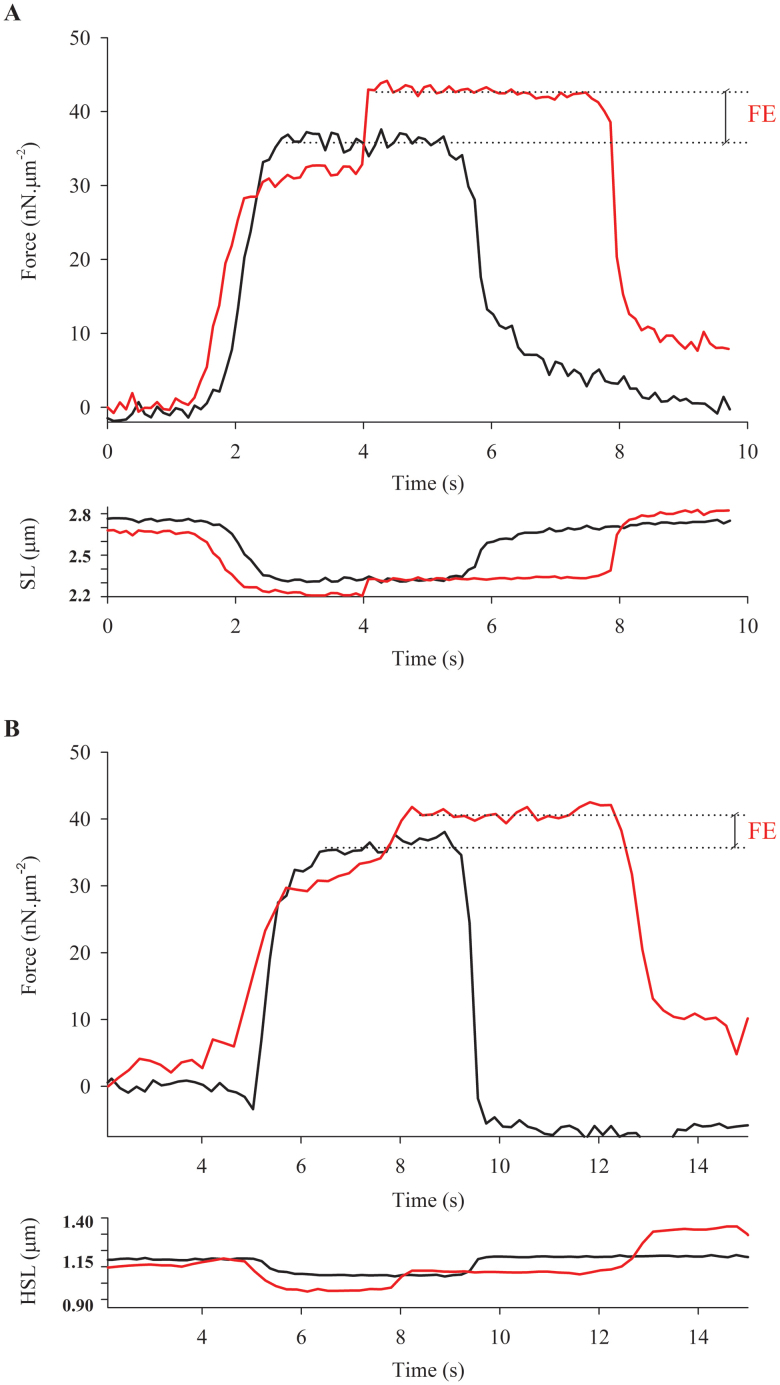
Typical experiment overview. A - Superimposed force traces (upper panel) and length traces (lower panel), from a single sarcomere activated in pCa^2+^ 4.5 and kept isometric (in black) or stretched during activation (in red). B - Superimposed force traces (upper panel) and length traces (lower panel), from a half-sarcomere activated in pCa^2+^ 4.5 and kept isometric (in black) or stretched (in red). In both cases, force enhancement (FE) was calculated as the difference between the isometric (in black) and steady state force (in red) achieved after stretch. Note that for the actual FE calculation, the passive component was also taken into account.

**Figure 2:**
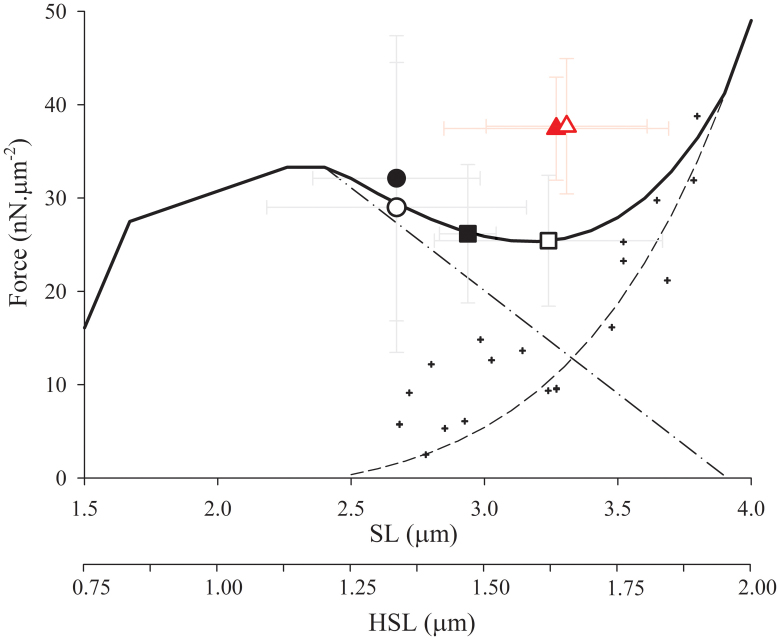
Mean force values (±SD) produced by single and half-sarcomeres. Predictive force-length relationship, constructed based on the filament lengths of rabbit psoas^13^. Mean isometric forces (±SD) produced by single (black closed symbols) and half-sarcomeres (black open symbols). The dashed line corresponds to exemplary passive force curves derived from two sets of experiments in relaxed sarcomeres (small crosses), the dash-dotted line corresponds to the descending limb of the force-length relationship, and the continuous line corresponds to the sum of the predictive force-length relationship to the passive curve. The mean forces after a stretch are shown in red for single sarcomeres (closed-triangle) and half sarcomeres (opened-triangle). The circles represent mean force values (±SD) from isometric contractions performed close to the plateau of the force-length relation, while the squares represent mean values (±SD) from isometric contractions performed at longer SL and HSL.

Figure 1 shows superimposed force traces obtained during an experiment with a single sarcomere (A), and a half-sarcomere (B). In both cases, the steady-state forces after stretch were higher than the force produced during the isometric contraction at the corresponding sarcomere lengths and half-sarcomere lengths. When we calculated the amount of force enhancement in our samples, passive forces were taken into account; the passive-tension-curve was derived from another set of sarcomeres that were passively stretched from ~2.5 to 4.0 um. Figure 2 shows the pooled data from both groups plotted over a predicted force-length curve based on the filament lengths of rabbit psoas^13^.

An ANCOVA model showed that, after adjusting for the amount of stretch applied to the sarcomeres and half sarcomeres, there was a significant difference in the average force produced after stretch when compared to the isometric contractions (p = 0.0004). However, we did not observe differences between the two groups (sarcomeres and half-sarcomeres) (p = 0.3). There was no interaction between the group (sarcomere, half-sarcomere) and the condition (isometric, after stretch), implying that the difference in mean force after stretch is independent of the preparation. The statistical results are shown in details in Tables 1 and 2. Note that this table summarizes only the experiments that were used in the ANCOVA analysis; outliers and experiments which were not completed are not presented in here.

## Discussion

This is the first study that shows the feasibility of mechanically isolating and testing half-sarcomeres from striated muscles. The preparation may open opportunities for future studies on the mechanics of striated muscles with far-reaching implications; the half-sarcomere is the smallest functional unit of muscle that contains all molecules in a three-dimensional intact lattice. The forces produced by the half-sarcomeres are within the range of those observed in larger preparations, including sarcomeres^7^,^8^,^12^, myofibrils^5^,^14^,^15^,^16^, and cells^17^,^18^,^19^. Most importantly, in the context of the current study, our results show that the force produced by the half-sarcomeres is significantly increased during stretch, and it remains enhanced after stretch. Although it is difficult to directly compare the forces after stretch with the force produced at precisely similar lengths with this preparation, our results indicate clearly that half-sarcomeres can produce a long-lasting force enhancement. When we compared the forces after stretch with forces produced at isometric contractions at relatively similar lengths, and most directly when we compare the same forces with the predicted force-length relation curve for isometric contractions based on the degree of filament overlap^20^ using the filament length from the rabbit psoas muscles^13^, they are clearly elevated. This result suggests a mechanism other than HSL non-uniformity in force enhancement.

Previously, we showed that single sarcomeres were able to produce residual force enhancement^8^. We attributed the phenomenon partially to A-band displacements that happen during activation and stretch. We suggested that force enhancement was caused partly by half-sarcomere length non-uniformities, and partly by passive components present within the half-sarcomeres, although we have not measured the mechanics of isolated half-sarcomeres. In the current study we confirmed that elements within the half-sarcomere can produce a stretch-induced force enhancement. The most likely mechanisms that can explain force enhancement in half-sarcomeres are an increase in the number of attached cross-bridges after stretch, and/or an increase in the stiffness of titin upon muscle activation^21^,^22^. Recent studies suggest that the increase in stiffness after stretch is in fact caused by an increase in the stiffness of titin^22^,^23^, which could also explain our results with half-sarcomeres. Titin molecules become stiffer upon Ca^2+^ binding^21^, which can lead to an increased force after stretch^22^.

Based on expected forces to be produced during isometric contractions, confirmed by the isometric forces that we measured in this study that falls into the well-defined force-length relation^20^, the stretch-induced increase in force is in the same order of magnitude as reported in previous studies^7^. However, our results are in stunning contradiction with a previous study using myofibrils, which found extremely large levels of force enhancement (>250%) after stretch^24^. In that study, myofibrils were stretched by approximately 40% SL along the descending limb of the force-length relation. We could not repeat such experiments; stretches of similar magnitude invariably causes irreversible damage to the preparations.

Although single sarcomere and half-sarcomere preparations offer innumerous advantages with a large potential for the study of molecular mechanics of striated muscles, they also have a few limitations when compared to larger preparations (i.e. single fibres and muscle bundles). First, the number of contractions that can be elicited with these preparations before a decrease in force is observed (likely due to damage) is reduced to three to four activations, which makes its use limited according to the goals of the study. Secondly, it is very difficult to control the amount of half-sarcomere shortening during activation and contractions. This limitation also makes difficult to control the actual magnitude of stretch that is imposed to the preparation during experiments. Lastly, inserting a micro-needle inside the preparations is invasive, and could interfere with its contractile properties. It could change the myosin-to-myosin filaments spacing altering the probability of myosin-actin interactions in certain regions of the half-sarcomere. Although we were not able to overcome these limitations, our results show force traces that not only resemble the behavior of larger preparations (activation and relaxation), but are also within the range of values of force produced by larger preparations, including sarcomeres^7^,^8^,^12^, myofibrils^5^,^14^,^15^,^16^, and cells^17^,^18^,^19^. Thus, we are confident that our results are reliable and represent the true contractile activity of the sarcomeres and half-sarcomeres.

In conclusion, we presented an experimental preparation that can be used to test the mechanics of the most basic contractile unit of striated muscles: the half-sarcomere. The forces produced by half-sarcomeres are similar to those produced by larger preparations, providing confidence that they are not damaged and can be used for solving a variety of issues in the field of muscle biophysics. The half sarcomeres produce a long-lasting increase in force after stretch, suggesting that sarcomeric components are involved in the residual force enhancement. We suggest that titin is such component – future work should test directly this hypothesis.

## Methods

### Preparation of the single and half-sarcomeres

Single and half sarcomeres were isolated from rabbit psoas muscle using a modified procedure explained previously by our group^7^. Shortly, small bundles of the muscles were tied to wooden sticks, and chemically permeabilized using a standard protocol^8^. The muscles were incubated in rigor solution (pH = 7.0) for approximately 4 hours, after which they were transferred to a rigor:glycerol (50:50) solution for 20 hours. The samples were placed in a new rigor:glycerol (50:50) solution with the addition of a mixture of protease inhibitors (Roche Diagnostics, USA) and stored in a freezer (−20°C) for at least seven days. The protocol was approved by the McGill University Animal Care Committee and complied with the guidelines of the Canadian Council on Animal Care.

On the day of the experiment, a muscle sample was transferred to a fresh rigor solution and stored in the fridge for one hour before use. A small section of the sample was extracted (~1 mm^3^) and homogenized in a rigor solution (pH = 7.0) using the following sequence: twice for 5 s at 7,500 rpm, and once for 3 s at 18,000 rpm. The homogenizing protocol produces a supernatant containing single myofibrils. This homogenate was transferred into an experimental chamber with the bottom made of a vacuum grease-sealed glass coverslip (thickness: 0.15 mm), placed on the stage of an inverted microscope (NIKON Eclipse TE 2000U). The chamber was filled with rigor solution, and the temperature was controlled at ~10°C with a circulating cooling solution running through a channel surrounding the chamber. The sample was rinsed several times, and after a rest period of 5 min, the rigor solution was slowly exchanged by a relaxing solution. A myofibril was chosen based on its striation appearance, and either a single sarcomere or a half sarcomere was selected for mechanical experimentation.

### Micro-needle production and calibration

The micro-needles were produced with a vertical pipette puller (KOPF 720, David Kopf Instruments) and calibrated by a cross-bending method^7^, using a pair of micro-fabricated cantilevers of known stiffness (489 and 592 nN/μm). The final stiffness of the micro-needles used during these experiments varied between 35 and 400 nN/μm.

### Mechanical isolation, visualization, and force measurement of single and half-sarcomeres

**Figure 3:**
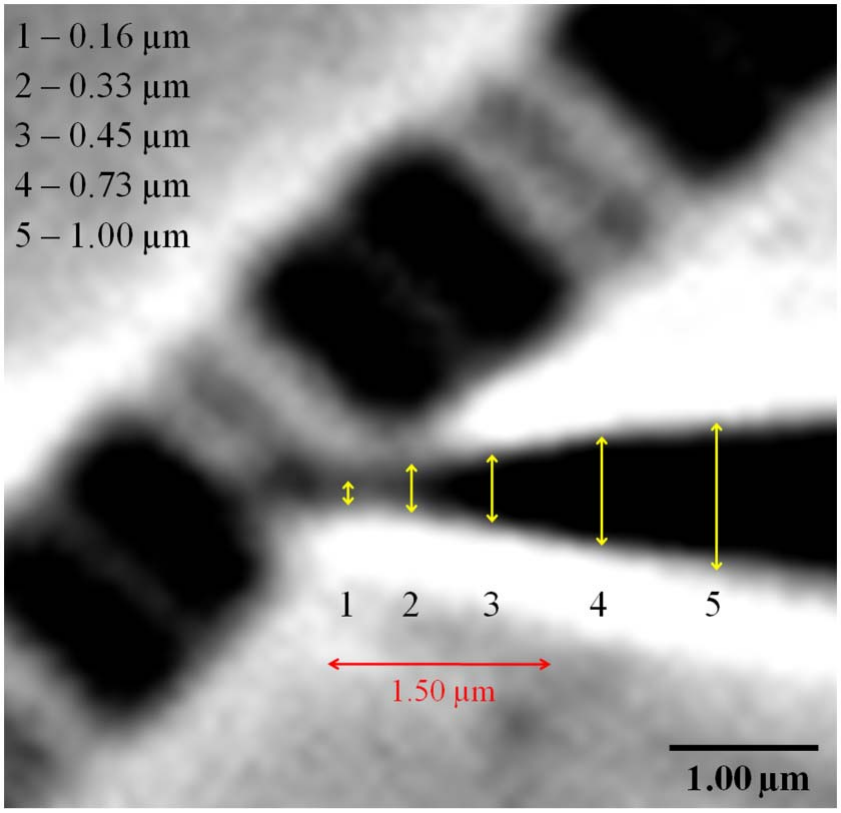
Needle dimensions. Tip of a glass micro-needle piercing a myofibril (on the left) externally adjacent to the Z-line from one of its sarcomeres. The figure shows representative measurements (on the top-left, yellow arrows) of five arbitrary cross-sectional areas of the conical-shaped tip of the needle. The red arrow represents how much of this tip was inserted into the myofibril; this value was never higher than 1.5 μm. Magnification = 150X.

**Figure 4:**
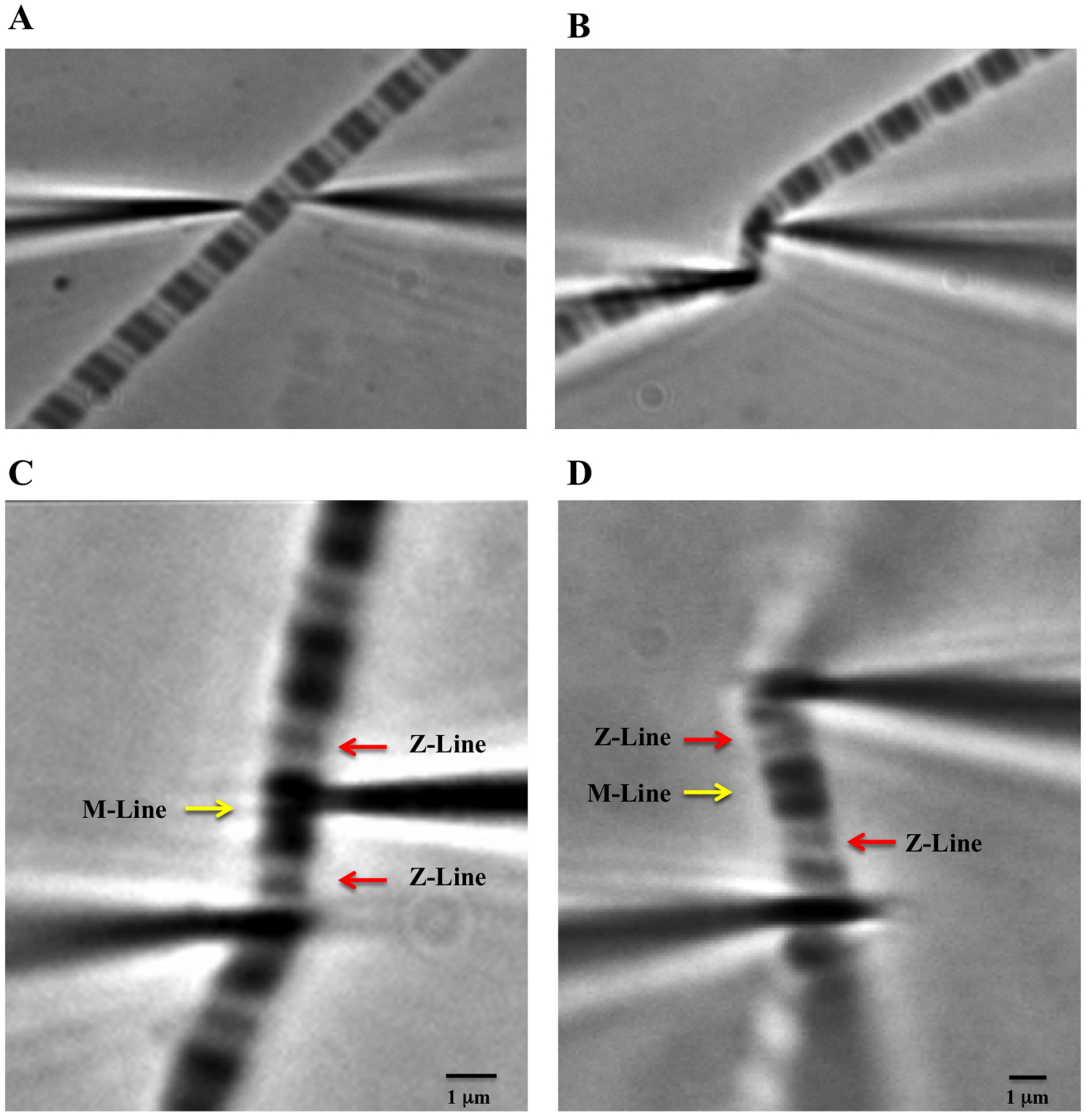
Half and single sarcomere isolation. A - Glass micro-needles approaching a single myofibril on the microscope coverslip (magnification = 90X). B - Half-sarcomere caught between the micro-needles. The myofibril is still on the coverslip (magnification = 90X). C - Half-sarcomere isolated and lifted (~2 μm) from the coverslip (magnification = 150X). D - Single sarcomere caught between two micro-needles (c).

Using micromanipulators (Narishige NT-88-V3, Tokyo, Japan), single sarcomeres or half-sarcomeres were captured by two pre-calibrated micro-needles (Figures 3 and 4). The dimensions of the micro-needles were similar to those used in our previous study using single sarcomeres; the needles present a conical-shaped tip, and only a small length (~1.5 μm, Figure 3 in red) is inserted into the myofibril. The diameters of the circular cross-sectional area of the portion of the cone inside the myofibril are normally bellow ~0.5–0.7 μm, while its tip is under 0.2 μm. The calibration of the micro-needles also followed a protocol established earlier in our laboratory^7^ and of others^25^. The needles were pierced externally adjacent to Z-lines in the case of sarcomeres (Figure 4D and Movie 1 suppl.), or between the Z-line and externally adjacent to the M-line (Figures 4B, 4C, and Movie 2 suppl.). The samples were raised from the glass coverslip by ~0.5-1.0 μm. Under high magnification provided by an oil immersion phase-contrast lens (Nikon plan-fluor, ×100, numerical aperture 1.30), the images of the single and half-sarcomeres were further magnified 1.5× by an internal microscope function. The contrast between the micro-needles produces a pattern of light intensity peaks that allow for tracking of their centroids using a particle tracker algorithm^26^. The half-sarcomere length was obtained by interpolating the displacement of the micro-needles from the initial to the final distances measured from the Z-line to the center of the sarcomere. The force produced during activation of the single and half-sarcomeres was obtained by measuring the displacement of the micro-needles, as described elsewhere^7^.

### Solutions

The rigor solution (pH 7.0) was composed of (in mM): 50 Tris, 100 NaCl, 2 KCl, 2 MgCl_2_, and 10 EGTA. The activating (pCa^2+^ of 4.5) and relaxing (pCa^2+^ of 9.0) - pH 7.0- solutions contained (in mM): 20 imidazole, 14.5 creatine phosphate, 7 EGTA, 4 MgATP, 1 free Mg^2+^, free Ca^2+^ in two concentrations adjusted to obtain pCa^2+^ of 4.5 (32 μM) and 9.0 (1 nM); KCl was used to adjust the ionic strength to 180 mM in all solutions.

### Protocol

Single sarcomeres (n = 18) and half-sarcomeres (n = 17) were immersed in relaxing solution for 1–2 s. The solution was rapidly replaced by an activating solution using a computer-controlled, multichannel perfusion system (VC-6M, Harvard Apparatus) and a double-barreled pipette^7^. When surrounded by the activating solution, the preparations contracted and produced force. Each experiment counted with 2–3 isometric contractions produced at different lengths, and a contraction in which a stretch was imposed to the preparation. For the isometric contractions, the sarcomeres and half sarcomeres were passively adjusted to a desired length before activation; the nominal lengths were chosen to be 2.7 μm and 3.0 μm for the two contractions. However, differently from experiments performed with larger preparations, activation of sarcomeres and half-sarcomeres produced varying degrees of shortening, which made it difficult to have the experiments at exactly the same lengths.

For the stretch contraction, after full force development was obtained, single sarcomeres and half-sarcomeres were stretched by different magnitudes, ranging from 15–36% of half-sarcomere length (HSL), at speeds ranging from 1.35 to 3.15 μm·s^−1^·HSL^−1^. After the end of the stretch, the myofibrils were held isometric for at least 5 s before relaxation. Nominal length changes induced to the preparation resulted in varying levels of actual sarcomere/half-sarcomere stretching, which made a precise pre-determination of the final lengths difficult. For each preparation we performed a series of passive stretches, starting at ~2.4 μm and ending at ~4.0 μm, with intervals of at least 10 s between stretches.

### Data analysis

The results of this study are reported as means ± standard deviations (SD), since all the data analyzed were normally distributed. To compare the mean isometric forces between sarcomeres and half-sarcomeres, we used T-tests for independent samples. A two-way, mixed model, analysis of covariance (ANCOVA) was used to compare averages of forces produced after stretch and during isometric contractions at similar conditions, as well as the two groups investigated in this study (sarcomeres and half-sarcomeres), after adjusting for the amount of stretch. The mixed model ANCOVA takes into account the possible correlation between repeated measures within each experiment because they were subjected to both conditions (isometric and after stretch), and enabled the amount of stretch, which may influence the levels of force enhancement, to be used as a covariate in the model, to more accurately assess the potential differences in forces. The assumption of homogeneity of slopes for the ANCOVA model was checked by introducing and testing interaction effect terms between both the groups (sarcomere and half-sarcomere) and the conditions (isometric, after stretch), and the covariate (amount of stretch). The assumptions of linearity, normality and equal variances of errors, as well as the presence of possible outliers, were explored with analysis of residuals. The statistical analyses were carried out with SAS software (version 9.2). All hypothesis tests were two-sided and performed at the 0.05 significance level.

## Author Contributions

Conceived and designed the experiments: F.C.M. and D.E.R. Performed the experiments: F.C.M. and B.M.B. Analyzed the data: F.C.M., D.E.R. and J.A.C. Contributed with reagents/materials/analysis tools: D.E.R. Manuscript writing: F.C.M., D.E.R., B.M.B., M.A.V. and J.A.C.

## Supplementary Material

Supplementary Information Movie 1

Supplementary Information Movie 2

## Figures and Tables

**Table 1:** Force produced by half-sarcomeres and single sarcomeres during isometric contractions, and contractions in which a stretch was imposed during full activation. The adjusted means are the results of adjusting the force values for the amount of stretch using the ANCOVA analysis

	Force (nN·μm^−2^) produced during isometric contraction		Force (nN·μm^−2^) produced after stretch	
	Mean (±SD)	Adjusted mean (±SE)	Mean (±SD)	Adjusted mean (±SE)
Half-sarcomere (n = 10)	24.7 ± 9.5	24.6 ± 3.6	37.7 ± 7.3	37.6 ± 2.1
Single sarcomere (n = 14)	30.8 ± 11.9	30.9 ± 3.0	37.4 ± 5.5	37.5 ± 1.8

**Table 2:** Results of the ANCOVA analysis when comparing the conditions (after stretch and during the isometric contraction) and the group (single sarcomeres and half-sarcomeres), adjusted for the amount of stretch. SE: Standard error of the mean difference. CI: Confidence interval of the mean difference

	Mean Difference	SE	Adjusted 95% CI
Force (n·μm^−2^) produced after stretch vs. during isometric contraction	9.8	2.4	(4.9, 14.7)
Force (n·μm^−2^) produced by half-sarcomeres vs. single sarcomeres	3.1	3.1	(3.3, 9.5)
